# The Application of Metabolomic Techniques in Research Investigating Neurodegenerative Diseases

**DOI:** 10.3390/metabo9120283

**Published:** 2019-11-20

**Authors:** Brian D. Green

**Affiliations:** School of Biological Sciences, Institute for Global Food Security, Queen’s University Belfast, Biological Sciences Building, Chlorine Gardens, Stranmillis, Northern Ireland BT9 5DL, UK; b.green@qub.ac.uk

We live in a world posing many new and different challenges for human health, and one such challenge is the rapidly expanding number of cases of human neurodegenerative disease. Many of the most common neurodegenerative diseases are dementias affecting cognitive and behavioural functions, and it is very concerning that treatment options remain extremely limited. The unmet medical needs for many conditions are extremely high because, unlike many of the other common non-communicable diseases (NCDs), such as cardiovascular diseases, cancers and diabetes, few disease-modifying therapies exist. The causes are multifactorial and the potential disease drivers are numerous. Aside from the rising age profile of the global population and the known genetic risk factors, there are many potential modifiable risk factors, ranging from hypertension, obesity, hearing loss, smoking, depression, physical inactivity, social isolation, diabetes and years of education [1]. The progressive and terminal nature of these conditions places a considerable personal burden on the individual affected. Additionally, there is a growing economic and public health burden, forcing governments and health services to make difficult choices concerning the allocation of medical resources. Tens of millions of people are indiscriminately affected by various dementias, which are rising at an alarming rate [2].

It has been emphasised that the quantity of basic science in dementia research lags behind many other diseases [3]. So in order to make progress here, our fundamental understanding of how biochemical processes are affected by these chronic, complex and seemingly stealthy diseases needs to improve. There is a need for new disease classification strategies and early diagnostic tools. Metabolomics still represents a relatively new field of analytical science, which can be extremely useful in the early diagnosis of disease. The relatively unique feature of metabolites is that they sit at the intersection between the genetic background of an organism and its environment. Since many neurodegenerative diseases are not genetically inherited (instead having a range of known genetic risk factors and also a large number of unknown environmental triggers), metabolomics offers great promise for the discovery of new, biologically and clinically relevant biomarkers for neurodegenerative disorders. It is already bringing forward new knowledge in terms of the mechanisms of neurodegenerative diseases. For instance, work of our own indicates that, viewed longitudinally, the metabolic impact of Alzheimer’s pathology is transient, perhaps with distinct phases [4], and is undoubtedly affected by severity [5]. The last 10 years of metabolomics research has brought forward a considerable amount of new biochemical knowledge about diseases such as Alzheimer’s disease (AD), however, many other diseases are underrepresented and new collaborations and initiatives are needed for metabolomics to better penetrate these research areas.

Overall, this Special Issue of Metabolites presents a collection of cutting-edge studies and review articles demonstrating the application of metabolomics for the investigation of neurodegenerative diseases. The issue covers a broad range of disease areas, including AD, Parkinson’s disease (PD), Cerebral palsy (CP) and age-related macular degeneration (AMD), but also includes conditions such as traumatic brain injury (TBI) and transient ischemic attacks (TIA). Within the research articles, metabolomic methods include ^1^H NMR, direct injection liquid chromatography-tandem mass spectrometry (DI/LC-MS/MS), gas chromatography-mass spectrometry (GC-MS) and LC-MS following perfusion with ^13^C-labelled compounds. There are also reviews of the different method types that can be utilised in neurodegenerative disease research, including imaging mass spectrometry (IMS) and direct mass spectrometry-based approaches. Finally, the articles feature the analysis and review of data from clinical samples, various rodent models and also more fundamental models such as *C. elegans.*

I hope that you enjoy reading this special issue.
